# Safety and efficacy of the maximum tolerated dose of givinostat in polycythemia vera: a two-part Phase Ib/II study

**DOI:** 10.1038/s41375-020-0735-y

**Published:** 2020-02-11

**Authors:** Alessandro Rambaldi, Alessandra Iurlo, Alessandro M. Vannucchi, Richard Noble, Nikolas von Bubnoff, Attilio Guarini, Bruno Martino, Antonio Pezzutto, Giuseppe Carli, Marianna De Muro, Stefania Luciani, Mary Frances McMullin, Nathalie Cambier, Jean-Pierre Marolleau, Ruben A. Mesa, Raoul Tibes, Alessandro Pancrazzi, Francesca Gesullo, Paolo Bettica, Sara Manzoni, Silvia Di Tollo

**Affiliations:** 1 Department of Oncology and Hematology, University of Milan and ASST Papa Giovanni XXIII, Bergamo, Italy; 2 Hematology Division, Fondazione IRCCS Ca’ Granda Ospedale Maggiore Policlinico, Milan, Italy; 3 Department of Experimental and Clinical Medicine, University of Florence, Azienda Ospedaliera-Universitaria Careggi, Florence, Italy; 4 Department of Hematology, Royal Cornwall Hospital, Truro, UK; 5 Department of Hematology, Oncology and Stem Cell Transplantation, Medical Center, Faculty of Medicine, University of Freiburg, Freiburg, Germany; 6 Department of Hematology and Oncology, Medical Center, University of Schleswig-Holstein, Campus Lübeck, Lübeck, Germany; 7 Hematology Unit, IRCCS Istituto Tumori “Giovanni Paolo II”, Bari, Italy; 8 Oncology-Hematology Department, A.O. “Bianchi-Melacrino-Morelli”, Reggio Calabria, Italy; 9 Department of Hematology Oncology, Charité’ Medical School, Campus Benjamin Franklin, Berlin, Germany; 10 U.O. Hematology, San Bortolo Hospital, Vicenza, Italy; 11 Hematology and Stem Cells Transplantation Unit, Campus Bio-Medico, University Hospital, Rome, Italy; 12 U.O. Clinical Hematology, Presidio Ospedaliero “Spirito Santo”—A.S.L. Azienda Sanitaria Locale, Pescara, Italy; 13 Centre for Medical Education, Queen’s University Belfast, Belfast, UK; 14 Service d’Oncologie Hématologie, Hospital Saint Vincent de Paul—GHICL Lille, Lille, France; 15 Service d’Oncologie Hématologie Clinique, CHU Amiens—Hospital Sud, Amiens, France; 16 Mayo Clinic Cancer Center, Scottsdale, AZ USA; 17 New York University School of Medicine & Perlmutter Cancer Center/ NYU Langone Health, New York, NY USA; 18 Clinical R&D Department, Italfarmaco S.p.A., Cinisello Balsamo, Italy

**Keywords:** Haematopoietic stem cells, Haematopoiesis

## To the Editor:

Polycythemia vera (PV) is a chronic myeloproliferative neoplasm (cMPN) characterized by stem cell-derived clonal myeloproliferation resulting in panmyelosis with persistently raised hematocrit, increased risk of thrombotic complications, and predisposition to evolve to myelofibrosis or leukemia [1]. Therapy is currently based on phlebotomy to normalize hematocrit, and aspirin. Hydroxyurea is used as first line when cytoreduction is necessary [1], although toxicity can result in inadequate disease management [2]. Recently, ropeginterferon α-2b was approved by European Medicinal Agency as first line for patients without symptomatic splenomegaly [3]. Ruxolitinib is second-line for patients who are refractory and/or intolerant to hydroxyurea [4]; other treatments include busulfan, pipobroman [5], and nonpegylated and pegylated interferons (off-label) [1, 6, 7], but use is limited by side effects and safety concerns. Additional, targeted therapies are therefore needed.

Up to 98% of patients with PV bear the *JAK2*^V617F^ gene mutation, which activates erythropoietin receptor signaling pathways. Givinostat is a histone-deacetylase (HDAC) inhibitor that selectively targets *JAK2*^V617F^ cell growth, reducing hematopoietic cell proliferation [8]. The efficacy and safety of givinostat alone or with hydroxyurea has previously been evaluated in two studies in *JAK2*^V617F^ positive PV [9, 10]. Although these studies confirmed the positive risk-benefit of givinostat, they did not provide comprehensive efficacy evidence for givinostat monotherapy, and did not identify the most appropriate dose. The current study was therefore conducted to support givinostat monotherapy development in PV, aiming to determine the maximum tolerated dose (MTD), and to assess safety and efficacy of this dose.

This multinational, open-label, nonrandomized study was conducted in two parts. *Part A* (Phase Ib) was dose escalation, with the first 4-week cycle determining the MTD. *Part B* (Phase II), the proof of concept phase, then evaluated efficacy and safety at this MTD. Full details of the methods are in the supplement. Both parts had 24-week treatment periods, with patients receiving six four-week cycles of givinostat. In *Part A*, since givinostat 50 mg twice daily (BID) was previously well tolerated, the first cohort of three patients received 100 mg BID, with the dose to be escalated by 50 mg BID in each subsequent cohort according to a 3 + 3 design, adopting a modified Fibonacci escalation scheme, although only after the third patient had been followed for a minimum of one cycle, and tolerability data had been evaluated by the Safety Review Team (Supplementary Table 1). For *Part B*, patients initially received givinostat at the MTD, with modification permitted to achieve an optimized dose, balancing tolerability, and response.

Eligible patients were aged ≥18 years, with a confirmed PV diagnosis, *JAK2*^V617F^ positivity assessed by centralized quantitative real-time polymerase chain reaction, and active/not controlled disease, defined as: (1) hematocrit ≥45% or <45% with phlebotomy, and (2) platelet count >400 × 10^9^/l, and (3) white blood cell count >10 × 10^9^/l. Main exclusion criteria were: absolute neutrophil count <1.2 × 10^9^/l; prior *JAK2* or HDAC inhibitor treatment; systemic treatment for cMPNs other than aspirin; hydroxyurea, interferon alpha, or anagrelide within 28, 14, or 7 days before enrollment, respectively. All patients provided informed consent. Study registration: ClinicalTrials.gov (NCT01901432).

The primary objectives of *Part A* were to determine givinostat’s MTD, and to characterize safety and tolerability in terms of treatment-related adverse events (AEs). Secondary endpoints were to evaluate overall response after three and six cycles (using the clinico-hematological European LeukemiaNet (ELN) response criteria [11]), and to characterize pharmacokinetics. For *Part B*, primary objectives were to evaluate overall response, safety and tolerability after three cycles. Secondary endpoints were to evaluate overall response, safety and tolerability after six cycles, and to characterize pharmacokinetics. Exploratory endpoints are in the supplement.

Twelve patients were studied in *Part A*, with 35 in *Part B* (Supplementary Fig. 1, Supplementary Tables 2 and 3). In *Part A*, during the first cycle one patient receiving givinostat 100 mg BID experienced dose-limiting toxicity: Grade 3 dyspepsia, drug related, resolving with sequelae after treatment. Three additional patients therefore received 100 mg BID, none of whom had dose-limiting toxicity during Cycle 1. Although escalation to higher doses was permitted, the Safety Review Team agreed the MTD was 100 mg BID, given: (a) thrombocytopenia is a known side effect of HDAC inhibitors; (b) a platelet count decrease was observed in subsequent cycles; (c) as givinostat is a chronic treatment, it was preferable to not expose patients to higher doses that could be poorly tolerated during chronic treatment. To more accurately define givinostat’s MTD, three additional patients received an intermediate dose (150 mg daily). Finally, three patients received 50 mg BID, to investigate safety, pharmacokinetics, and pharmacodynamics of this dose. A total of 66.7% of patients experienced at least one drug-related AE, mainly Grade 1 or 2 (Supplementary Table 4), most commonly thrombocytopenia (33.3% of patients). Two patients (16.7%) experienced a serious AE (thrombophlebitis and myocardial infarction), neither drug-related; no patient died. Two patients withdrew due to drug-related AEs (dyspepsia [Grade 3] and thrombocytopenia [Grade 4]), both with 100 mg BID.

**Fig. 1:**
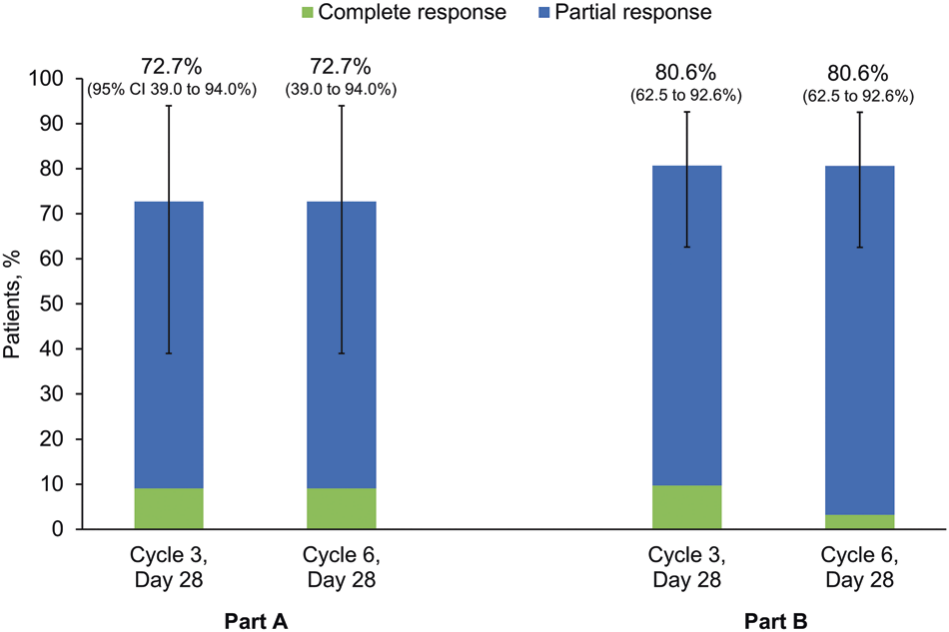
Parts A and B: therapeutic response evaluation (intention-to-treat population). Data are from 11 patients in Part A and 31 patients in Part B.

The overall response rate in *Part A* was above 70% (Fig. 1). One patient achieved complete response after three cycles, and one after six cycles. Median givinostat *T*_max_ was 1.5–4 h (Supplementary Table 5), with steady-state reached by day 28 of Cycle 1 (the first repeat-dose pharmacokinetic evaluation). After three cycles, givinostat normalized hematological parameters in 45.5–54.5% of patients (Supplementary Table 6), normalized spleen volume in 54.5%, resolved disease-related symptoms in 63.6% (Supplementary Table 7), and reduced pruritus and *JAK2*-mutated allele burden (Supplementary Table 8).

At the end of Cycles 3 and 6 of *Part B*, 80.6% of patients were responders (Fig. 1), with three achieving complete response after three cycles and one after six. Overall, 94.3% of patients had at least one drug-related AE, the majority Grade 1 or 2 and none Grade 4 or 5, with most occurring during the first three cycles of treatment (152 out of 190 events). The most common were diarrhea (51.4% of patients), thrombocytopenia (45.7%), and increased blood creatinine (37.1%) (Table 1). Two patients experienced a serious AE, both during the first three cycles, one study drug-related (Grade 3 diarrhea resolving in 7 days without therapy, with study drug temporarily discontinued). Three patients withdrew, one due to study drug-related AEs (Grade 3 neutropenia and Grade 2 thrombocytopenia, both resolving). The other two were withdrawn by their investigators (Supplementary Fig. 1). No patient died, and there were no clinically relevant vital signs or ECG values.

**Table 1:** Part B: patients with study drug-related treatment-emergent AEs, overall and by system organ class and preferred term (including only preferred terms reported by one or more patient with Grade 3 events) (safety population).

System organ class preferred term	Grade 3		Any grade	
	*N*	%	*N*	%
Patients with any drug-related AE	10	28.6	33	94.3
Blood and lymphatic system disorders	3	8.6	18	51.4
Anemia	2	5.7	6	17.1
Neutropenia	1	2.9	2	5.7
Thrombocytopenia	1	2.9	16	45.7
Cardiac disorders	0^a^	0	1	2.9
Gastrointestinal disorders	4	11.4	26	74.3
Diarrhea	4	11.4	18	51.4
General disorders and administration site conditions	2	5.7	9	25.7
Asthenia	2	5.7	8	22.9
Investigations	0^a^	0	19	54.3
Metabolism and nutrition disorders	1	2.9	8	22.9
Hypocalcemia	1	2.9	4	11.4
Nervous system disorders	0^a^	0	5	14.3
Renal and urinary disorders	0^a^	0	2	5.7
Respiratory, thoracic and mediastinal disorders	0^a^	0	1	2.9
Skin and subcutaneous tissue disorders	1	2.9	6	17.1
Rash	1	2.9	1	2.9

Data are from 35 patients. Grades are based on the National Cancer Institute Common Terminology Criteria for Adverse Events Version 4.03, where Grade 1 are mild events, Grade 2 are moderate, Grade 3 are severe, Grade 4 are life-threatening, and Grade 5 events result in death. There were no Grade 4 or 5 events in Part B of the study.

*AE* adverse event.

^a^There were no Grade 3 events for these system organ classes.

Overall, *Part B* pharmacokinetics was similar to *Part A* at comparable doses (Supplementary Tables 9 and 10). Improvements were seen in all individual response criteria (Supplementary Fig. 2; Supplementary Table 11), with white blood cell and platelet counts normalized in 90.3% and 74.2% of patients after three cycles, respectively, and hematocrit in 77.4% and 48.4% after three and six cycles, respectively. Improvements were observed in disease-related symptoms assessed by Myeloproliferative Neoplasm Symptom Assessment Form quality of life (QoL) questionnaire, especially during Cycle 6 (Supplementary Table 12), with a reduction in the proportion with severe pruritus (Score 7–10; Supplementary Fig. 3). Approximately 50% had no headache (Supplementary Table 12), and no patients had severe headache (Supplementary Fig. 4). The proportion of patients without microvascular symptoms improved from baseline (38.7%) to Cycle 6 (51.6%; Supplementary Table 12), with a low proportion having severe symptoms (6.5–12.9%; Supplementary Fig. 5). A total of 19.4% had a spleen volumetric index reduction of at least 35% during treatment, with total spleen normalization in two and three patients after three and six cycles, respectively, and a moderate reduction in *JAK2*^V617F^ allele burden (Supplementary Table 12). Finally, differential gene expression was observed (Supplementary Fig. 6), with upregulation for *GLRX*, *STAT4* and *HDAC3*, and downregulation for *MYC*.

The study aims were achieved, with the MTD, 100 mg BID, determined in *Part A*, and this dose effective in *Part B*. In addition to the high overall response rate, givinostat had a positive impact on individual clinico-hematological ELN criteria, both hematological parameters and disease-related symptoms. The three hematological parameters, all abnormal at study entry, were normalized in the majority of patients, and givinostat improved key disease-related symptoms, notably pruritus with complete resolution in many patients, with an associated positive impact on QoL. A reduction in JAK2-mutated allele burden was observed in both parts of the study, and *Part B* provided clear evidence of differential gene expression with givinostat, consistent with disease pathway regulation. Overall, givinostat was well tolerated with no new safety concerns. Unlike previous studies, the recruited population had active or not controlled disease, and were both high- and low-risk, making comparisons difficult. However, the observed response was greater than for other PV therapies [12–15]. For example, in a study comparing interferon to hydroxyurea, 45% of patients had a hematologic response to either therapy [12], whereas in a second study, 40% of patients had a response of any type to ruxolitinib [13], and in a third the overall response to the HDAC inhibitor vorinostat was 35%, with significant side effects resulting in a high rate of study withdrawal [15].

In conclusion, these data support givinostat monotherapy development in the defined PV target population.

### Supplementary information

Supplemental material
