# Supplementary material for: Safety and efficacy of the maximum tolerated dose of givinostat in polycythemia vera: a two-part Phase Ib/II study
Source: Leukemia. 2020 Feb 11;34(8):2234–7. doi: 10.1038/s41375-020-0735-y (PMC7387307; doi:10.1038/s41375-020-0735-y)
Supplement: Supplementary file 1 — Supplemental material [file 41375_2020_735_MOESM1_ESM.pdf]

# Safety and efficacy of the maximum tolerated dose of givinostat in polycythemia vera: A two-part Phase Ib/II study

Alessandro Rambaldi, Alessandra Iurlo, Alessandro M. Vannucchi, Richard Noble, Nikolas von Bubnoff, Attilio Guarini, Bruno Martino, Antonio Pezzutto, Giuseppe Carli, Marianna De Muro, Stefania Luciani, Mary Frances McMullin, Nathalie Cambier, Jean-Pierre Marolleau, Ruben A. Mesa, Raoul Tibes, Alessandro Pancrazzi, Francesca Gesullo, Paolo Bettica, Sara Manzoni, and Silvia Di Tollo

## Supplemental information

# Methods

## *Study design*

For ***Part A***, therapeutic response, spleen size (by magnetic resonance imaging [MRI] or computed tomography [CT]) and disease-related symptoms (using the Myeloproliferative Neoplasm Symptom Assessment Form [MPN-SAF] quality of life [QoL] questionnaire) were evaluated on Day 28 of Cycles 3 and 6, with blood samples taken for the quantitative real-time polymerase chain reaction (qRT-PCR) evaluation of *JAK2*$^{V617F}$ mutational status on peripheral blood granulocytes. Blood chemistry and hematology samples were collected, and electrocardiogram (ECG) assessments were performed regularly throughout Cycle 1, and on Day 28 of all subsequent cycles, with ECG evaluations also performed on Day 2 of Cycle 1.

For ***Part B***, therapeutic response, spleen size, and disease-related symptoms were evaluated on Day 28 of Cycles 3 and 6. Blood chemistry, hematology, *JAK2*$^{V617F}$ qRT-PCR and ECG evaluations were performed on Day 28 of each cycle.

## *Part A dose selection*

The study safety review team comprised an independent expert clinician, the study principal investigator, and staff from the sponsor and contract research organization. The protocol permitted intermediate dose levels to be introduced to more accurately define the maximum tolerated dose (MTD). After the MTD was determined, a cohort of patients was also recruited to receive givinostat 50 mg BID for pharmacokinetic (PK) profiling.

No intra-patient dose escalation was permitted prior to determining the MTD of givinostat. Subsequently, patients who experienced dose-limiting toxicity were permitted dose adjustment, with their givinostat dose reduced up to twice.

*Supplemental Table 1. Rules applied to determine the maximum tolerated dose*

| **Number of patients with dose-limiting toxicity at a given dose level** | **Action** |
|---|---|
| None out of three | Enter three patients at the next dose level. |
| One out of three | Enter at least three more patients at this dose level and:<br>• If none of these three patients experiences dose-limiting toxicity, proceed to the next dose level.<br>• If at least one of this group experiences dose-limiting toxicity (i.e., at least two of the six patients has dose-limiting toxicity), this dose exceeds the maximum tolerated dose and dose escalation is stopped.<br>To further assess tolerability, three additional patients will be entered at the next lowest dose level if only three patients were treated previously at that dose level. Upon determination of the maximum tolerated dose, the study will proceed directly to Part B. |
| Two or more | Dose escalation will be stopped. This dose exceeds the maximum tolerated dose.<br>To further assess tolerability, three additional patients will be entered at the next lowest dose level if only three patients were treated previously at that dose level, and the study will proceed directly to Part B. |
| Dose-limiting toxicity was defined as the following drug-related toxicities, not related to disease progression or concomitant illnesses:<br>• Grade 4 hematological toxicities, or<br>• Grade 3 febrile neutropenia, or<br>• Grade ≥3 non-hematological toxicities with the exception of:<br>  o Grade 3 diarrhea without adequate supportive care lasting less than three days, and<br>  o Grade 3 nausea or vomiting without adequate supportive care lasting less than three days, or<br>• any drug-related serious adverse event (AE), or<br>• any toxicity that was clearly not related to disease progression or intercurrent illness requiring interruption of dosing for more than three days during the first cycle.<br>Grades were based on the National Cancer Institute Common Terminology Criteria for Adverse Events (NCI CTCAE) Version 4.03, where Grade 3 is severe, and Grade 4 is life-threatening. | |

### *Exploratory endpoints*

Exploratory endpoints in both parts of the study were to evaluate the effects of givinostat on: individual parameters of the response criteria;[1] PD markers; spleen size in patients with confirmed splenomegaly at baseline; pruritus, headache and microvascular symptoms according to the MPN-SAF QoL questionnaire;[2] *JAK2*$^{V617F}$ allele burden; and the reduction in symptomatic treatment of pruritus. In ***Part B*** only, as an exploratory endpoint, the efficacy of givinostat was assessed after six cycles using the *revised* ELN response criteria,[3] both overall and using each individual parameter.

The spleen size evaluation was performed by imaging (i.e., MRI or CT), according to institutional guidelines and site-specific clinical practice, with the same technique and instrument to be used throughout the study. The MPN-SAF QoL questionnaire is a 27-item validated instrument, with each item marked on a scale of 0 to 10, where 1 is most and 10 is least favorable.[2] The *JAK2*$^{V617F}$ allele burden was assessed by qRT-PCR and a standard curve using the WHO First International Reference Panel for Genomic *JAK2*$^{V617F}$ (Version 3.0, Dated 13/06/2018),[4] with mutation presence and allele burden evaluated in triplicate in each sample. The impact of givinostat on the symptomatic treatment of pruritus was evaluated in terms of dose and/or days of treatment of medication used to treat this symptom. Finally, molecular markers were measured for the PD evaluation by a central laboratory.

## Sample size and statistical methods

For ***Part A***, sample size was based a 3+3 design, adopting a modified Fibonacci escalation scheme.[5–7] For ***Part B***, Simon's two-stage design[8] was employed to test the null hypothesis that the overall response rate would be ≤ 50% against the alternative hypothesis that it would be ≥ 75%, for which 28 patients eligible for the primary endpoint had to be enrolled.

The number and percentage of patients responding to treatment was calculated with the associated two-tailed 95% confidence interval (CI) computed using the normal

approximation or exact method (as applicable). Plasma PK calculations for givinostat and its metabolites were performed by a central laboratory using standard non-compartmental analysis using Phoenix WinNonlin 6.3 (Certara Company, Princeton, NJ, USA).

The safety population included all enrolled patients who received at least one dose of study drug. The intention-to-treat (ITT) population included all recruited patients who received at least one dose of study drug and from whom at least one post-baseline efficacy measurement was obtained. In order to assess the robustness of the efficacy analysis, the analysis of the efficacy endpoints was repeated in the per protocol (PP) population that included all ITT patients who received at least 14 daily doses of study drug without interruption, and who did not experience any major protocol deviation (in both parts of the study, efficacy analyses performed on the PP analysis set supported the ITT population results). The MTD population included all patients who experienced dose-limiting toxicity in Cycle 1 of ***Part A***, or who received ≥ 90% of the doses of study drug in Cycle 1 of ***Part A***. The PK population comprised all patients with at least one PK assessment.

## Results

***Part A*** was conducted between October 2013 and February 2016. Of 21 patients screened, 12 received at least one dose of givinostat (six assigned to givinostat 100 mg BID, three to 50 mg BID, and three to 100 mg in the morning and 50 mg in the evening), with nine (75%) completing all six cycles of treatment. The three patients who did not complete the study were all assigned to givinostat 100 mg BID: two withdrew due to AEs (both considered study drug-related; see the main text for details), and one withdrew consent.

One patient assigned to givinostat 100 mg BID did not provide any post-baseline efficacy data, and so was excluded from the ITT and PP populations. Five further patients were excluded from the PP population due to protocol deviations. No patients were excluded from the MTD or PK populations.

***Part B*** was conducted between December 2015 and September 2017. Of 44 patients screened, 36 were enrolled, with 35 receiving at least one dose of medication, 31 of whom completed three cycles of treatment, and 27 completed the study (Supplemental Figure 1). Three patients withdrew, one due to study drug-related AEs (Grade 3 neutropenia and Grade 2 thrombocytopenia, both resolving; Supplemental Table 2). The other two patients were withdrawn by their investigators, and had the following AEs ongoing at the end of the study: one had Grade 1 controlled hypertension (not study drug-related, did not resolve), Grade 1 anemia (study drug-related, resolved with sequelae), and Grade 1 alopecia (study drug-related, did not resolve); the other patient had Grade 2 asthenia (study drug-related, resolved without therapy), Grade 2 deep vein thrombosis (not study drug-related, treated with concomitant therapy and resolved), and Grade 3 pulmonary embolism (not study drug-related, treated with concomitant therapy and resolved).

## *Tables*

*Supplemental Table 2. Neutrophil and white blood cell count in the patient withdrawn from Part B due to study drug-related adverse events*

| | Baseline | End of Cycle 1 | End of Cycle 2 | UNSCH | End of Cycle 3 | UNSCH | UNSCH | UNSCH | End of study |
|---|---|---|---|---|---|---|---|---|---|
| Neutrophil ($10^9$/l) | 9.8 | 2.9 | 1.7 | 2.2 | 0.8 | 0.3 | 1.6 | 2.1 | 2.7 |
| White blood cells ($10^9$/l) | 11.8 | 5 | 4.3 | 4.6 | 2.3 | 3.5 | 3.8 | 3.9 | 4.7 |

UNSCH, data from an unscheduled visit.

*Supplemental Table 3. Baseline demographics and disease characteristics (safety population)*

| | Part A (N = 12) | Part B (N=35) |
|---|---|---|
| Age, years, median (range) | 62.5 (51 to 77) | 58.0 (39 to 80) |
| Age group, number (%) | | |
| < 60 years | 3 (25.0) | 19 (54.3) |
| ≥ 60 years | 9 (75.0) | 16 (45.7) |
| Sex, number (%) | | |
| Male | 7 (58) | 24 (69) |
| Female | 5 (41) | 11 (31) |
| Race, white, number (%) | 12 (100) | 35 (100) |
| Time since diagnosis, months, mean ± SD | 78.4±63.53 | 63.2±74.43 |
| High-risk patients, number (%)* | 9 (75.0) | 16 (45.7) |
| ≥ 60 years | 9 (75.0) | 15 (42.9) |
| History of previous thrombosis | 0 | 0 |
| Both criteria | 0 | 1 (2.9) |
| Hematology, median (range) | | |
| Hemoglobin, g/l | 132.5 (121 to 160) | 134.0 (118 to 194) |
| Hematocrit, % | 45.2 (41.0 to 48.0) | 45.9 (40.9 to 56.7) |
| Platelets, $10^9$/l | 703.0 (403 to 1332) | 740.0 (446 to 1459) |
| White blood cells, $10^9$/l | 15.10 (10.90 to 46.48) | 15.58 (10.27 to 30.10) |
| Spleen | | |
| Patients with splenomegaly, number (%) | 7 (58.3) | 27 (77.1) |

| | Part A (N = 12) | Part B (N=35) |
|---|---|---|
| Volumetric index, $cm^3$, median (range)† | 48.56 (19.63 to 190.06) | 45.84 (16.23 to 273.78) |
| Longitudinal diameter, cm, median (range) | 14.85 (8.0 to 29.7) | 14.80 (7.0 to 35.0) |
| Patients with, number (%) | | |
| Pruritis | 11 (91.7) | 28 (80.0) |
| Headache | 7 (58.3) | 17 (48.6) |
| Microvascular symptoms | 7 (58.3) | 22 (62.9) |
| *JAK2*$^{V617F}$ allele burden, %, median (range) | 70.85 (43.5 to 89.5) | 73.50 (33.4 to 94.2) |
| Prior therapy for polycythemia vera, number (%) | | |
| At least one prior disease medication | 10 (83.3) | 30 (85.7) |
| Antithrombotic treatment | | |
| Acetylsalicylic acid | 5 (41.7) | 26 (74.3) |
| Cytoreductive treatments | | |
| Hydroxyurea | 7 (58.3) | 16 (45.7) |
| Interferon | 2 (16.7) | 2 (5.7) |
| Fedratinib | 0 | 1 (2.9)‡ |

*Derived data, based on patient's age and history of thrombosis reported in the 'Medical History' case report form. †Splenic volumetric index is calculated as (longitudinal diameter × antero-posterior diameter × transversal diameter) divided by 27. ‡This was a formal protocol deviation (violating one of the exclusion criteria), but was permitted after discussion with the sponsor (the patient ceased taking fedratinib more than two years prior to study entry, and the sponsor's authorization took into account the half-life of fedratinib, the patient's medical need, and that the patient's profile was in line with the study protocol population); no post-baseline efficacy data were obtained, and so this patient is not included in the intention-to-treat population.

*Supplemental Table 4. Part A: Patients with study drug-related treatment-emergent AEs, overall and by system organ class and preferred term (safety population)*

| **System organ class** | **Grade 3 or 4** | | **Any grade** | |
|---|---|---|---|---|
| **Preferred term** | **N** | **%** | **N** | **%** |
| **Patients with any drug-related AE** | 4 | 33.3 | 8 | 66.7 |
| Blood and lymphatic system disorders | 3 | 25.0 | 4 | 33.3 |
| Hemolytic anemia | 0 | 0 | 1 | 8.3 |
| Thrombocytopenia | 3 | 25.0 | 4 | 33.3 |
| Gastrointestinal disorders | 1 | 8.3 | 4 | 33.3 |
| Abdominal pain | 0 | 0 | 1 | 8.3 |
| Diarrhea | 0 | 0 | 1 | 8.3 |
| Dry mouth | 0 | 0 | 2 | 16.7 |
| Dyspepsia | 1 | 8.3 | 1 | 8.3 |
| Feces soft | 0 | 0 | 1 | 8.3 |
| General disorders and administration site conditions | 0 | 0 | 3 | 25.0 |
| Asthenia | 0 | 0 | 2 | 16.7 |
| Early satiety | 0 | 0 | 1 | 8.3 |
| Fatigue | 0 | 0 | 1 | 8.3 |
| Investigations | 0 | 0 | 3 | 25.0 |
| Blood creatinine increased | 0 | 0 | 1 | 8.3 |
| Blood lactate dehydrogenase increased | 0 | 0 | 1 | 8.3 |
| Weight decreased | 0 | 0 | 1 | 8.3 |
| Metabolism and nutrition disorders | 0 | 0 | 1 | 8.3 |
| Decreased appetite | 0 | 0 | 1 | 8.3 |
| Nervous system disorders | 0 | 0 | 4 | 33.3 |
| Dysgeusia | 0 | 0 | 3 | 25.0 |
| Headache | 0 | 0 | 2 | 16.7 |
| Memory impairment | 0 | 0 | 1 | 8.3 |
| Psychiatric disorders | 0 | 0 | 1 | 8.3 |
| Confusional state | 0 | 0 | 1 | 8.3 |

| System organ class<br>Preferred term | Grade 3 or 4 | | Any grade | |
|---|---|---|---|---|
| | N | % | N | % |
| Irritability | 0 | 0 | 1 | 8.3 |
| Renal and urinary disorders | 0 | 0 | 2 | 16.7 |
| Acute kidney injury | 0 | 0 | 1 | 8.3 |
| Chronic kidney disease | 0 | 0 | 1 | 8.3 |
| Skin and subcutaneous tissue disorders | 0 | 0 | 1 | 8.3 |
| Pruritus | 0 | 0 | 1 | 8.3 |

AE, adverse event. Data are from 12 patients. Grades are based on the National Cancer Institute Common Terminology Criteria for Adverse Events Version 4.03, where Grade 1 are mild events, Grade 2 are moderate, Grade 3 are severe, Grade 4 are life-threatening, and Grade 5 events result in death. There were no Grade 5 events.

*Supplemental Table 5. Part A: Plasma pharmacokinetic parameters of givinostat and its metabolites after single and repeat doses (pharmacokinetic population)*

| Parameter, unit | Givinostat | ITF2374 | ITF2375 | Givinostat | ITF2374 | ITF2375 |
|---|---|---|---|---|---|---|
| | **Givinostat 50 mg twice a day** | | | | | |
| | **Cycle 1, Day 1 (N=3)** | | | **Cycle 1, Day 28 (N=3)** | | |
| $T_{max}$, h | 2.00 | N/A | N/A | 3.90 | N/A | 2.00 |
| $C_{max}$, ng/ml | 60.2±43.1 | N/A | N/A | 22.4±8.92 | N/A | 110±22.8 |
| Geometric mean ratio vs Day 1 | | | | 0.484 (0.184 to 1.27) | N/A | N/A |
| $T_{last}$, h | 7.97±0.05 | N/A | N/A | 8.05±0.249 | N/A | 3.0±1.41 |
| $AUC_{last}$, ng.h/ml | 208±136 | N/A | N/A | 132±45.1 | N/A | 263±192 |
| Geometric mean ratio vs Day 1 | | | | 0.731 (0.342 to 1.56) | N/A | N/A |
| $AUC_{0\text{-}12h}$, ng.h/ml | 235±146 | N/A | N/A | 161±51.8 | N/A | 863 (N=1) |
| Geometric mean ratio vs Day 1 | | | | 0.762 (0.398 to 1.46) | N/A | N/A |
| | **Givinostat 100 mg in the morning and 50 mg in the evening** | | | | | |
| | **Cycle 1, Day 1 (N=3)** | | | **Cycle 1, Day 28 (N=3)** | | |
| $T_{max}$, h | 3.00 | 8.00 | 2.50 | 2.05 | 2.00 | 4.00 |
| $C_{max}$, ng/ml | 82.5±24.4 | 5.69±2.87 | 101±24.5 | 290±330 | 31.3±4.95 | 376±215 |
| Geometric mean ratio vs Day 1 | | | | 2.36 (0.898 to 6.22) | 5.90 (2.48 to 14.0) | 2.56 (2.08 to 3.16) |
| $T_{last}$, h | 8±0.0 | 8±0.0 | 8.0±0.0 | 8.02±0.0252 | 8.02±0.0252 | 6.70±2.30 |
| $AUC_{last}$, ng.h/ml | 429±109 | 29.6±9.64 | 611±146 | 1100±1050 | 158±42.6 | 1780±1190 |
| Geometric mean ratio vs Day 1 | | | | 1.97 (0.925 to 4.22) | 5.39 (1.95 to 14.9) | 1.85 (0.912 to 3.77) |
| $AUC_{0\text{-}12h}$, ng.h/ml | 508±107 | N/A | 870±182 | 1180±1050 | N/A | 2340±970 |
| Geometric mean ratio vs Day 1 | | | | 1.84 (0.959 to 3.52) | N/A | 2.18 (1.44 to 3.29) |

| Parameter, unit | Givinostat | ITF2374 | ITF2375 | Givinostat | ITF2374 | ITF2375 |
|---|---|---|---|---|---|---|
| | Givinostat 100 mg twice a day | | | | | |
| | Cycle 1, Day 1 (N=5) | | | Cycle 1, Day 28 (N=4) | | |
| $T_{max}$, h | 2.00 | 8.00 | 3.00 | 1.50 | 8.00 | 1.99 |
| $C_{max}$, ng/ml | 54.3±17.2 | 3.74±4.09 | 68.6±21.2 | 73.3±31.9 | 19.7±7.24 | 259±75.9 |
| Geometric mean ratio vs Day 1 | | | | 1.32 (0.599 to 2.89) | 4.56 (1.92 to 10.8) | 3.33 (2.87 to 3.86) |
| $T_{last}$, h | 8.0±0.0 | 8.0±0.0 | 8.0±0.0 | 7.0±2.01 | 7.0±2.01 | 7.0±2.01 |
| $AUC_{last}$, ng.h/ml | 238±55.6 | 14.4±19.0 | 416±153 | 359±203 | 102±11.7 | 1390±675 |
| Geometric mean ratio vs Day 1 | | | | 1.36 (0.728 to 2.52) | 6.93 (2.51 to 19.1) | 2.96 (1.77 to 4.95) |
| $AUC_{0-12h}$, ng.h/ml | 289±68.9 | N/A | 598±259 | 533±223 | N/A | 2020±1000 |
| Geometric mean ratio vs Day 1 | | | | 1.75 (0.980 to 3.13) | N/A | 2.88 (2.06 to 4.02) |

$T_{max}$, time to maximal concentration; N/A, not applicable; $C_{max}$, maximal plasma concentration; $T_{last}$, time to last detectable concentration; $AUC_{last}$, area under the plasma concentration–time curve up to the last detectable concentration; $AUC_{0-12h}$, area under the plasma concentration–time curve over the dosing interval. Data are mean ± standard deviation, except $T_{max}$ which is median and geometric mean ratio (90% confidence interval). $AUC_{last}$ and $AUC_{0-12h}$ were calculated using the linear trapezoidal rule. Geometric mean accumulation ratios and related 90% CIs were determined on log-transformed data using analysis of variance (ANOVA) models that contained terms for dose, day and dose·day, subject (daily dose) and it was considered as a random effect.

*Supplemental Table 6. Part A: Individual hematological parameters of the clinico-hematological ELN response criteria (intention-to-treat population)*

| **Parameter** | **Timepoint** | | |
|---|---|---|---|
| | **Baseline** | **Cycle 3, Day 28** | **Cycle 6, Day 28** |
| Hematocrit < 45% without phlebotomy | 0 | 6 (54.5) | 3 (27.3) |
| Platelet count ≤ $400 \times 10^9$/l | 0 | 5 (45.5) | 5 (45.5) |
| White blood cell count ≤ $10 \times 10^9$/l | 0 | 6 (54.5) | 2 (18.2) |

ELN, European LeukemiaNet. Data are the number (%) of patients. N=11.

*Supplemental Table 7. Part A: Individual spleen and symptom parameters of the clinico-hematological ELN response criteria (intention-to-treat population)*

| **Parameter** | **Timepoint** | | |
|---|---|---|---|
| | **Baseline** | **Cycle 3, Day 28** | **Cycle 6, Day 28** |
| Normal spleen size | 5 (41.7) | 6 (54.5) | 6 (54.5) |
| No disease-related symptoms* | Not available | 7 (63.6) | 8 (72.7) |

ELN, European LeukemiaNet. Data are the number (%) of patients. N=11. *Patients with no pruritus, no headache, and no microvascular disturbances, as indicated by a score of 0 for all three items in the Myeloproliferative Neoplasm Symptom Assessment Form quality of life questionnaire.

*Supplemental Table 8. Part A: Disease-related symptoms, as assessed with the MPN-SAF QoL Questionnaire, and $JAK2^{V617F}$ (intention-to-treat population)*

| **Parameter** | **Timepoint** | | |
|---|---|---|---|
| | **Baseline** | **Cycle 3, Day 28** | **Cycle 6, Day 28** |
| Headache | | | |
| Mean score* | 1.5±1.83 | 1.7±2.87 | 0.9±1.36 |
| Mean percent change from baseline* | | 16.7±88.2% | –20.4±49.8 |
| None† | 5 (45.5%) | 5 (45.5%) | 6 (54.5%) |
| Microvascular symptoms | | | |
| Mean score* | 2.8±3.13 | 1.9±2.80 | 1.8±2.64 |
| Mean percent change from baseline* | | 1.8±67.9% | –11.1±34.4% |
| None† | 4 (36.4%) | 4 (36.4%) | 5 (45.5%) |
| Pruritis | | | |
| Mean score* | 4.9±3.32 | 2.6±3.00 | 2.3±2.65 |
| Mean percent change from baseline* | | 3.2±117.4% | –6.6±97.1% |
| None† | 1 (9.1%) | 3 (27.3%) | 2 (18.2%) |
| Overall QoL | | | |
| Mean score* | 2.5±2.43 | 2.8±2.59 | 1.6±1.59 |
| Mean percent change from baseline* | | 116.5±281.5% | 25.7±152.1% |
| Total score | | | |
| Mean score* | 64.2±42.12 | 44.6±41.80 | 41.2±30.35 |
| Mean percent change from baseline* | | –16.3±45.1% | –13.0±45.6% |
| $JAK2^{V617F}$ allele burden | | | |
| Absolute | 72.9±12.99% | 62.4±17.45% | 67.7±20.15% |
| Change from baseline | | –9.6±12.88% | –3.6±18.02% |

MPN-SAF, Myeloproliferative Neoplasm Symptom Assessment Form; QoL, quality of life. Data are mean ± standard deviation or the number (%) with no symptoms. N=11. *Evaluated by the MPN-SAF QoL questionnaire. †As indicated by a score of 0 for the associated item in the MPN-SAF QoL questionnaire.

*Supplemental Table 9. Part B: Plasma pharmacokinetic parameters of givinostat and its metabolites after a single dose of givinostat 100 mg twice a day (pharmacokinetic population)*

| **Parameter, unit** | **Givinostat** | **ITF2374** | **ITF2375** |
|---|---|---|---|
| $T_{max}$, h | 2.00 | 8.00 | 3.00 |
| $C_{max}$, ng/ml | 71.5±34.4 | 7.85±4.89 | 161±72.9 |
| $T_{last}$, h | 7.42±1.61 | 7.42±1.61 | 8.0±0.0505 |
| $AUC_{last}$, ng.h/ml | 289±130 | 28.5±14.0 | 888±439 |
| $AUC_{0\text{-}12h}$, ng.h/ml | 372±137 | N/A | 1080±619 |

$T_{max}$, time to maximal concentration; $C_{max}$, maximal plasma concentration; $T_{last}$, time to last detectable concentration; $AUC_{last}$, area under the plasma concentration–time curve up to the last detectable concentration; $AUC_{0\text{-}12h}$, area under the plasma concentration–time curve over the dosing interval; N/A, not applicable. Data are from 34 patients. Data are mean ± standard deviation, except $T_{max}$ which is median and geometric mean ratio (90% confidence interval).

*Supplemental Table 10. Part B: Repeat-dose plasma pharmacokinetic parameters of givinostat and its metabolites on Day 28 of Cycle 2 (pharmacokinetic population)*

| **Parameter, unit** | **Givinostat** | **ITF2374** | **ITF2375** | **Givinostat** | **ITF2374** | **ITF2375** |
|---|---|---|---|---|---|---|
| | **Givinostat 100 mg twice a day (N=17)** | | | **Givinostat 75 mg twice a day (N=11)** | | |
| $T_{max}$, h | 2.00 | 4.00 | 2.00 | 2.00 | 3.04 | 2.00 |
| $C_{max}$, ng/ml | 90.8±33.5 | 32.3±21.0 | 320±238 | 64.0±22.6 | 22.6±11.2 | 203±78.7 |
| Geometric mean ratio vs Cycle 1, Day 1 | 1.31 (1.09 to 1.58)* | 4.43 (3.65 to 5.36)* | 1.91 (1.58 to 2.31)* | NA | NA | NA |
| $T_{last}$, h | 8.00±0.0340 | 8.00±0.0340 | 7.75±1.00 | 7.98±0.0753 | 7.98±0.0753 | 8.00±0.00 |
| $AUC_{last}$, ng.h/ml | 459±145 | 216±127 | 1830±1660 | 323±107 | 161±83.5 | 1210±585 |
| Geometric mean ratio vs Cycle 1, Day 1 | 1.65 (1.38 to 1.96)* | 7.84 (6.19 to 9.92)* | 1.94 (1.61 to 2.35)* | NA | NA | NA |
| $AUC_{0\text{-}12h}$, ng.h/ml | 561±176 | NA | 2460±2450 | 410±129 | NA | 1460±608 |

Data are mean ± standard deviation, except $T_{max}$ which is median and geometric mean ratio (90% confidence interval). NA, not applicable. Geometric mean accumulation ratios and related 90% CIs were determined on log-transformed data using ANOVA that contained terms for day and subject was considered as a random effect, and dose-normalized data are presented. *Dose normalized data.

*Supplemental Table 11. Part B: Individual spleen and symptom parameters of the clinico-hematological ELN response criteria (intention-to-treat population)*

| **Parameter** | **Timepoint** | | |
|---|---|---|---|
| | **Baseline** | **Cycle 3, Day 28** | **Cycle 6, Day 28** |
| Normal spleen size | 5 (16.1) | 5 (16.1) | 5 (16.1) |
| No disease-related symptoms* | Not available | 23 (74.2) | 19 (61.3) |

ELN, European LeukemiaNet. Data are the number (%) of patients. N=31. *Patients with no pruritus, no headache, and no microvascular disturbances, as indicated by a score of 0 for all three items in the MPN-SAF QoL questionnaire.

*Supplemental Table 12. Part B: Disease-related symptoms, as assessed with the MPN-SAF QoL Questionnaire, and JAK2$^{V617F}$ (intention-to-treat population)*

| Parameter | Timepoint | | |
|---|---|---|---|
| | **Baseline** | **Cycle 3, Day 28** | **Cycle 6, Day 28** |
| Headache | | | |
| Mean score* | 1.9±2.35 | 0.8±1.37 | 1.0±1.39 |
| Mean percent change from baseline* | | –13.8±72.01% | –4.4±71.25% |
| None† | 17 (54.8%) | 18 (58.1%) | 15 (48.4%) |
| Microvascular symptoms | | | |
| Mean score* | 2.8±2.86 | 1.8±2.23 | 1.7±2.59 |
| Mean percent change from baseline* | | 19.2±127.52% | –3.8±129.46% |
| None† | 12 (38.7%) | 12 (38.7%) | 16 (51.6%) |
| Pruritis | | | |
| Mean score* | 4.9±3.73 | 2.0±2.68 | 2.0±2.64 |
| Mean percent change from baseline* | | –50.0±50.02% | –44.1±51.78% |
| None† | 6 (19.4) | 13 (41.9%) | 12 (38.7%) |
| Overall QoL | | | |
| Mean score* | 3.7±2.12 | 3.9±2.32 | 3.4±2.06 |
| Mean percent change from baseline* | | 19.4±111.59% | 26.8±166.43% |
| Total score | | | |
| Mean score* | 72.1±49.29 | 58.8±40.69 | 58.0±42.31 |
| Mean percent change from baseline* | | 1.4±87.74% | –10.1±49.19% |
| *JAK2*$^{V617F}$ allele burden | | | |
| Absolute | 68.8±18.01% | 48.9±24.55% | 54.1±23.33% |
| Change from baseline | | –17.2±18.12% | –11.6±17.14% |

MPN-SAF, Myeloproliferative Neoplasm Symptom Assessment Form; QoL, quality of life. Data are mean ± standard deviation or the number (%) with no symptoms. Data on symptoms and quality of life are for 31 patients in the ITT population; data on JAK2$^{V617F}$ allele burden are for 35 patients in the Safety population. *Evaluated by the MPN-SAF QoL questionnaire. †As indicated by a score of 0 for the associated item in the MPN-SAF QoL questionnaire.

## *Figures*

*Supplemental Figure 1. Part B: Patient disposition*

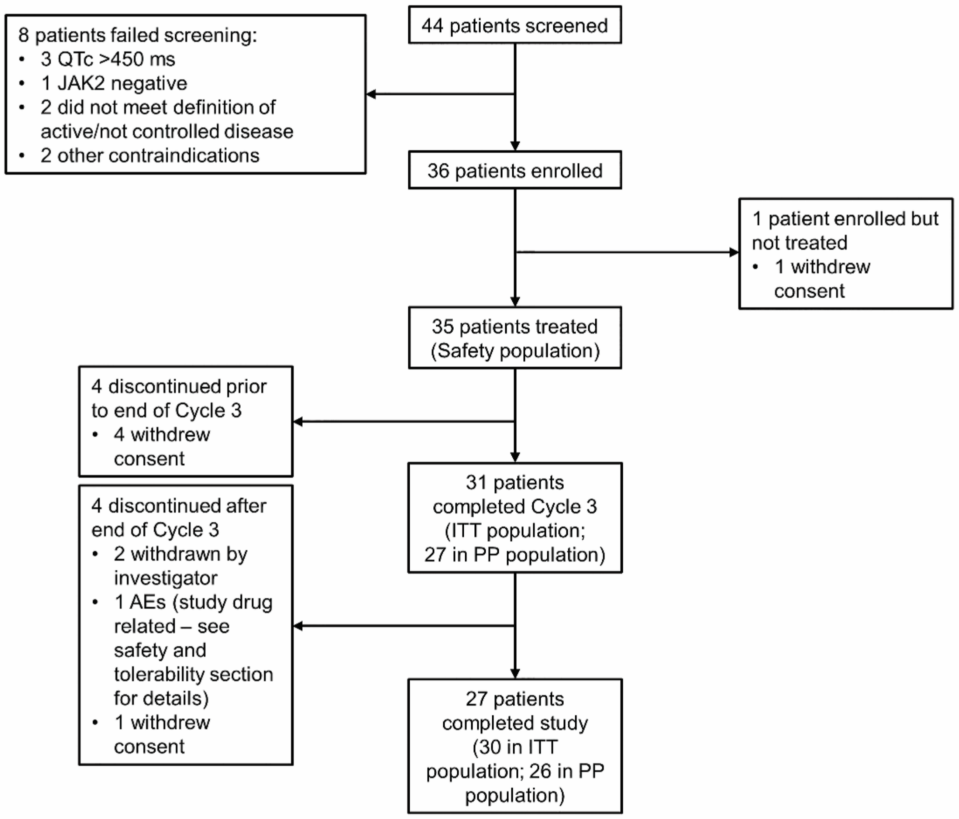


ITT, intention-to-treat; PP, per protocol; AE, adverse events.

*Supplemental Figure 2. Part B: Individual hematological parameters of the clinico-hematological ELN response criteria (intention-to-treat population)*

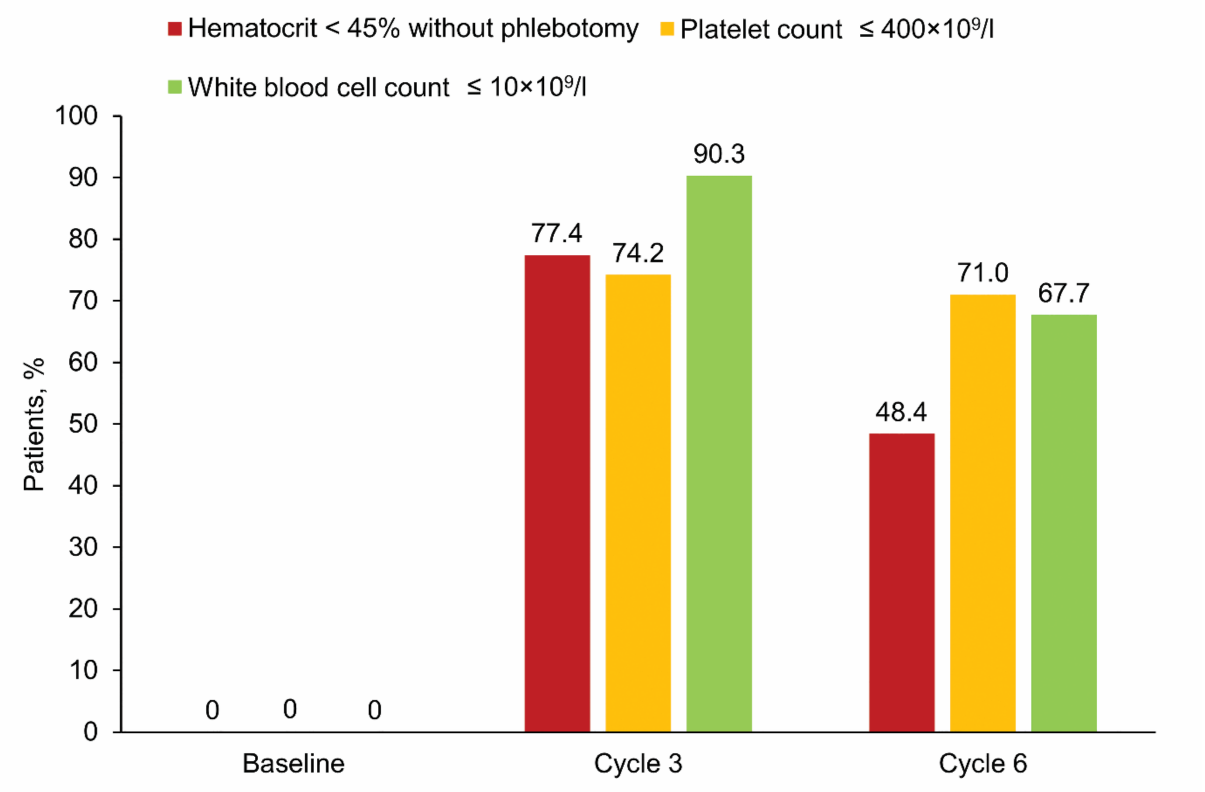


ELN, European LeukemiaNet. Data are from 31 patients. For Panel B, a score of 0 indicates that the patient was not experiencing the symptom at the timepoint.

*Supplemental Figure 3. Part B: Categorized pruritus symptom score (intention-to-treat population)*

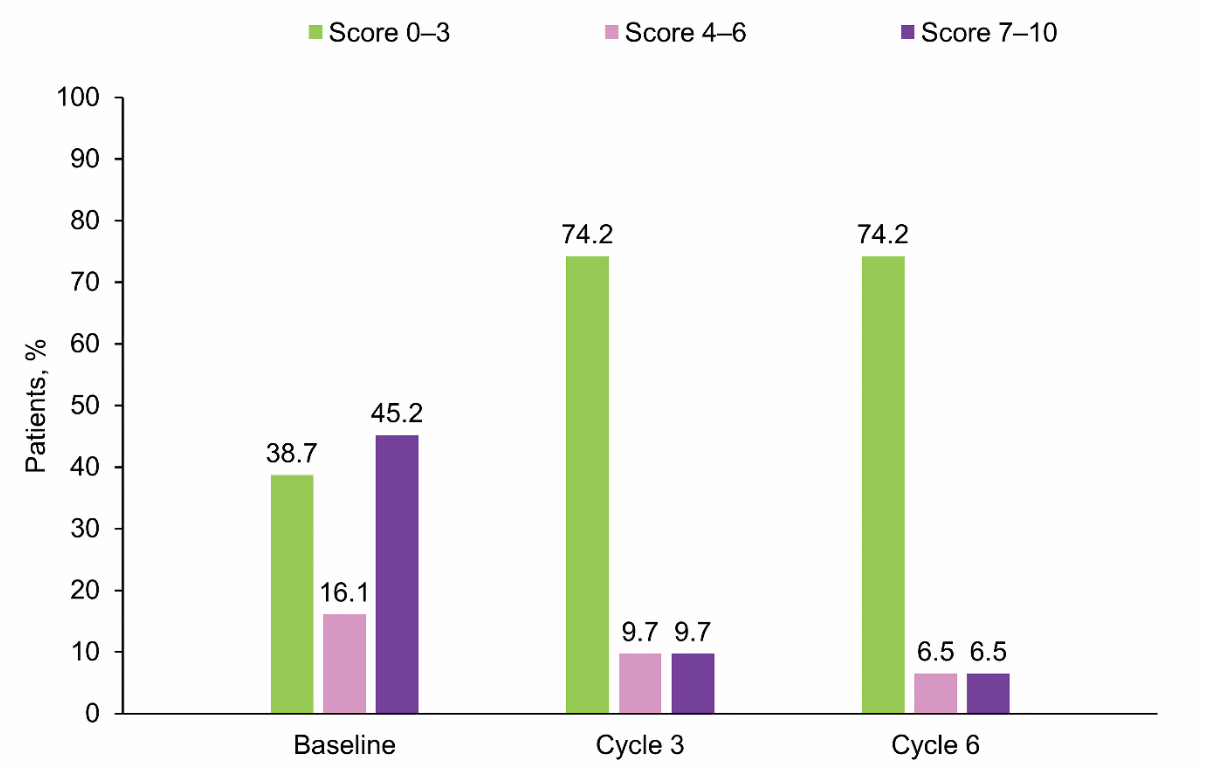


ELN, European LeukemiaNet. Data are from 31 patients. For Panel B, a score of 0 indicates that the patient was not experiencing the symptom at the timepoint.

*Supplemental Figure 4. Part B: Categorized headache symptom score (intention-to-treat population)*

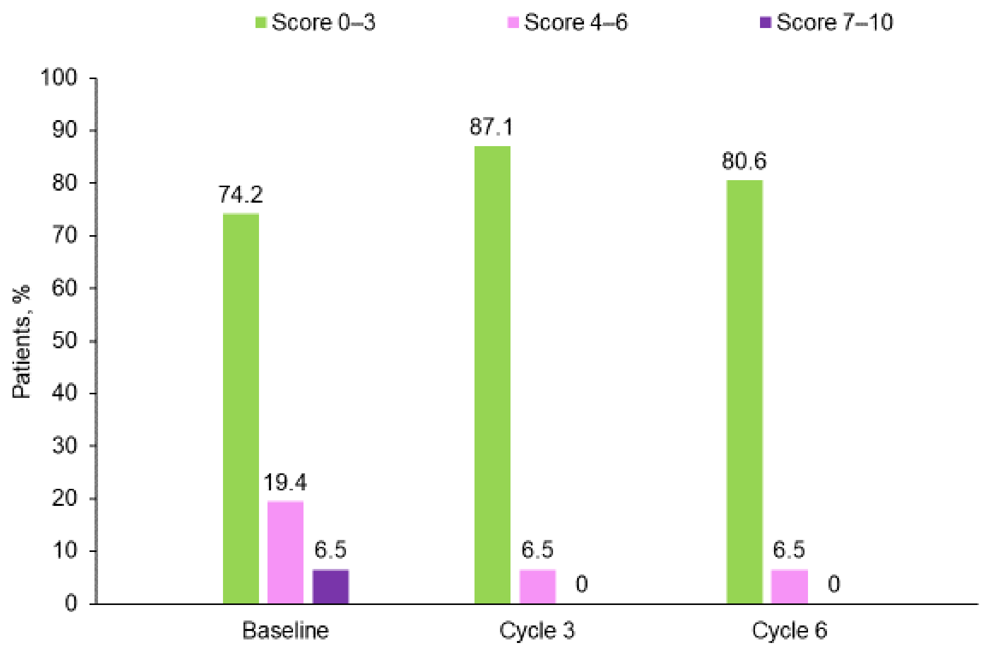


Data are from 31 patients. A score of 0 indicates that the patient was not experiencing the symptom at the timepoint.

*Supplemental Figure 5. Part B: Categorized microvascular symptom score (intention-to-treat population)*

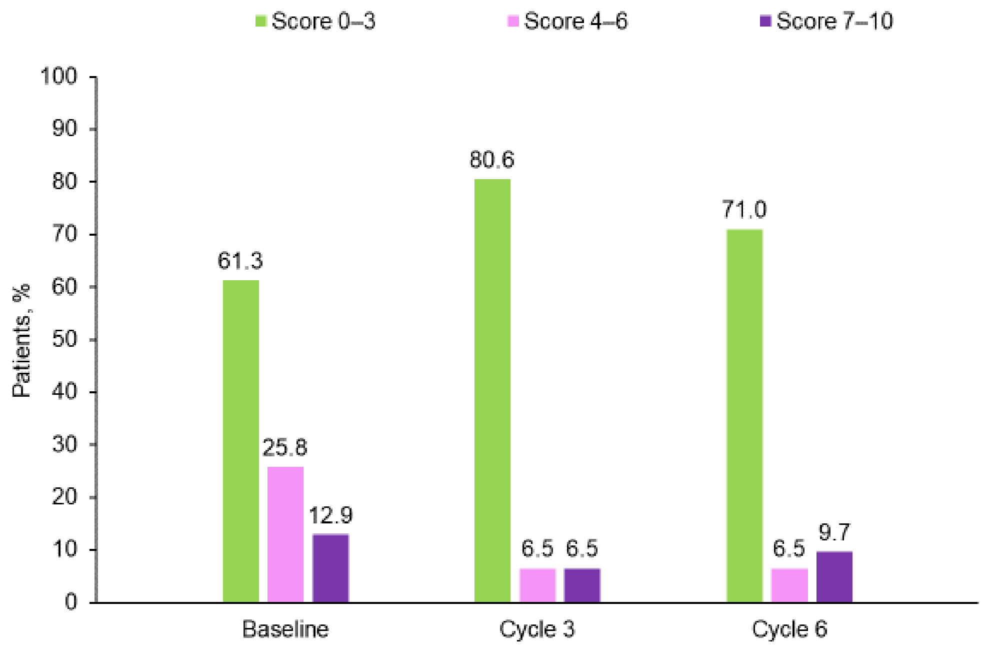


Data are from 31 patients. A score of 0 indicates that the patient was not experiencing the symptom at the timepoint.

*Supplemental Figure 6. Part B: Pharmacodynamic markers (intention-to-treat population)*

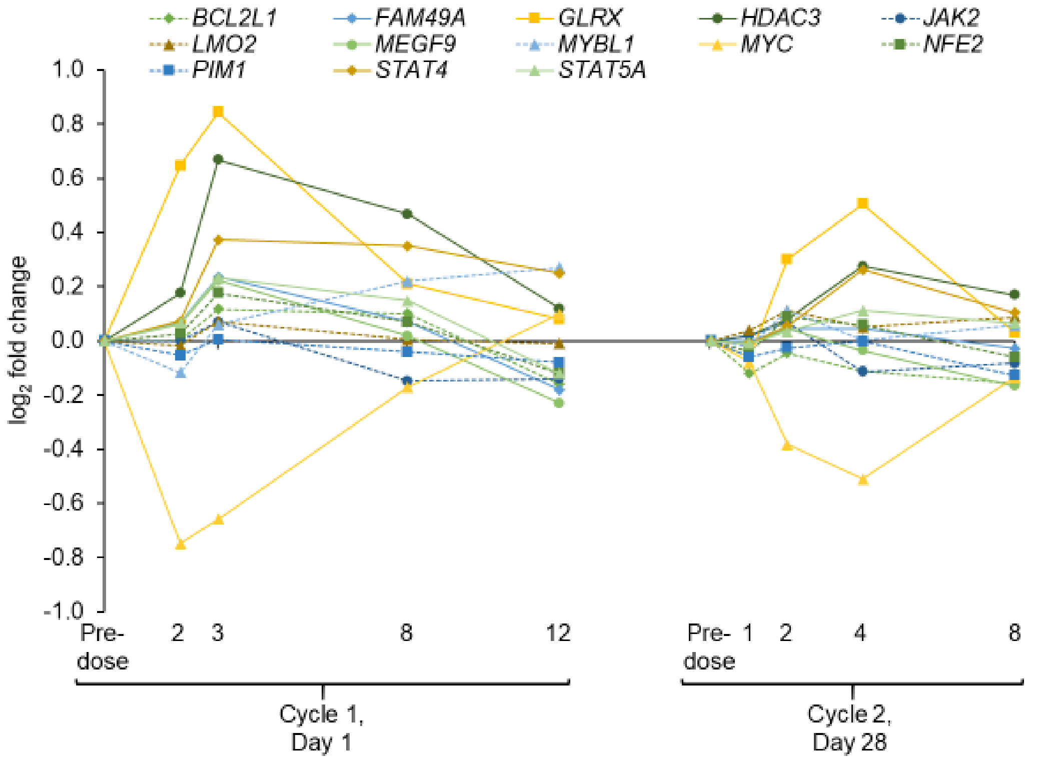


Data are changes in mean gene expression from the baseline sample (pre-dose) All timepoints relate to heparin tubes, except for 12-h post-dose in Cycle 1 which is PAXgene tube results. Genes with solid lines show at least one timepoint significantly different during Cycle 1 for both PAXgene and heparin samples. A value of +1 corresponds to a two-fold increase, and a value of –1 corresponds to a two-fold decrease. The intention-to-treat population comprised 31 patients. GLRX protects cells from DNA damage-inducing agents[9], STAT4 is required for interleukin-12-stimulated development of T helper 1[10], HDAC3 is involved in inflammatory gene expression[11], and MYC plays a role in cell cycle progression, apoptosis and cellular transformation[12].

## *Additional results, efficacy*

In ***Part B***, for the *revised* ELN response criteria[3] ten patients gave permission for bone marrow analyses, eight of whom (80%) met criteria for partial remission after six cycles, with none having either complete remission or progressive disease. None had any hemorrhagic or thrombotic event, two (20%) had durable resolution of disease-related signs; six (60%), eight (80%) and nine (90%) had durable response on the hematocrit, platelet, and white blood cell criteria, respectively, with two (20%) having bone marrow histological remission.

### *Additional results, safety*

Mean (SD) plasma creatinine values were as follows:

- Part A: 80.0 (20.2), 138.7 (37.7) and 99.2 (27.6) µmol/l at baseline and the end of Cycles 3 and 6, respectively.
- Part B: 83.9 (17.3), 105.3 (24.4) and 108.2 (22.3) µmol/l at baseline and the end of Cycles 3 and 6, respectively.

Note that no patients withdrew from the study due to blood chemistry abnormalities, and all AEs reported as ‘blood creatinine increased’ were Grade 1 or 2, with none reported as serious AEs in either part of the study.

## List of ethics committees

France

- Central ethics committee: Comité de Protection de Personnes Ile de France VI, Paris (03/07/2013).

Germany

- Central ethics committee: Ethik-Kommission an der TU Dresden, Dresden (23/10/2013).

Italy

- Azienda Socio Sanitaria Territoriale Papa Giovanni XXIII, Bergamo (29/05/2013).
- Comitato Etico per la Sperimentaz Clinica dei Medicinali AOU, Firenze (27/10/2014).
- Azienda Ospedaliero Universitaria Pisana, Pisa (24/07/2013).
- Comitato Etico per le attività biomediche dell'Università degli Studi Federico II, Napoli (04/11/2013).
- Comitato Etico Azienda Ospedaliera Bianchi Melacrino Morelli di Reggio Calabria, Reggio Calabria (08/01/2014).
- Instituto Tumori Giovanni Paolo II IRCCS Ospedale Oncologico Bari, Bari (12/11/2013).
- Comitato Etico Ospedale San Bortolo di Vicenza, Vicenza (31/12/2013).
- Comitato Etico Indipendente Locale, Bari (31/05/2016).
- Comitato Etico Referente per l'area di Pavia, Pavia (16/05/2016).
- Comitato Etico Milano Area 2, Milano (07/02/2017).
- Comitato Etico delle province di Chieti e Pescara, Chieti (12/05/2016).
- Università Campus Bio-Medico di Roma, Roma (12/04/2016).

Poland

- Central ethics committee: Komisja Bioetyczna przy Slaskiej Izbie Lekarskiej w Katowicach, Katowice (17/02/2014).

United Kingdom

- Central ethics committee: Health and Social Care Research Ethics Committee 3, Lisburn (17/09/2013).

## References


1 Barosi G, Rosti V. Novel strategies for patients with chronic myeloproliferative disorders. *Curr Opin Hematol* 2009; **16**: 129–34.

2 Scherber R, Dueck AC, Johansson P, Barbui T, Barosi G, Vannucchi AM *et al.* The Myeloproliferative Neoplasm Symptom Assessment Form (MPN-SAF): international prospective validation and reliability trial in 402 patients. *Blood* 2011; **118**: 401–8.

3 Barosi G, Mesa R, Finazzi G, Harrison C, Kiladjian J-J, Lengfelder E *et al.* Revised response criteria for polycythemia vera and essential thrombocythemia: an ELN and IWG-MRT consensus project. *Blood* 2013; **121**: 4778–81.

4 Sanzone P, Hawkins R, Rigsby P, Boyle J. Collaborative study to evaluate the proposed WHO 1st International Reference Panel for Genomic JAK2 V617F. Geneva, Switzerland: World Health Organization; 2016.

5 Rubinstein L, Simon R. Phase I clinical trial design. In: Budman, Calvert, Rowinsky, eds. *Handbook of Anticancer Drug Development*. Amsterdam: Elsevier; 2003.

6 Omura GA. Modified Fibonacci search. *J Clin Oncol* 2003; **21**: 3177–3177.

7 Le Tourneau C, Lee JJ, Siu LL. Dose escalation methods in phase I cancer clinical trials. *J Natl Cancer Inst* 2009; **101**: 708–20.

8 Simon R. Optimal two-stage designs for phase II clinical trials. *Control Clin Trials* 1989; **10**: 1–10.

9 Pandya P, Braiman A, Isakov N. PICOT (GLRX3) is a positive regulator of stress-induced DNA-damage response. *Cell Signal* 2019; **62**: 109340.

10 Thieu VT, Yu Q, Chang H-C, Yeh N, Nguyen ET, Sehra S *et al.* Signal Transducer and Activator of Transcription 4 Is Required for the Transcription Factor T-bet to Promote T Helper 1 Cell-Fate Determination. *Immunity* 2008; **29**: 679–690.

11 Angiolilli C, Kabala PA, Grabiec AM, Van Baarsen IM, Ferguson BS, García S *et al.* Histone deacetylase 3 regulates the inflammatory gene expression programme of rheumatoid arthritis fibroblast-like synoviocytes. *Ann Rheum Dis* 2017; **76**: 277–285.

12 Amati B, Land H. Myc-Max-Mad: a transcription factor network controlling cell cycle progression, differentiation and death. *Curr Opin Genet Dev* 1994; **4**: 102–8.
